# Editorial: Novelties in acute myeloid leukemia: from biology to clinical applications

**DOI:** 10.3389/fonc.2024.1499272

**Published:** 2024-10-17

**Authors:** Luca Vincenzo Cappelli, Nico Gagelmann, Hassan Awada, Carmelo Gurnari

**Affiliations:** ^1^ Department of Translational and Precision Medicine, Sapienza University of Rome, Rome, Italy; ^2^ Department of Stem Cell Transplantation, University Medical Center Hamburg-Eppendorf, Hamburg, Germany; ^3^ Roswell Park Comprehensive Cancer Center, Buffalo, NY, United States; ^4^ Department of Biomedicine and Prevention, University of Rome Tor Vergata, Rome, Italy; ^5^ Department of Translational Hematology and Oncology Research, Cleveland Clinic, Taussig Cancer Institute, Cleveland, OH, United States

**Keywords:** acute myeloid leukemia, prognostic biomarker, oncogenes, single cell omics, chemotherapy, CAR T cell, targeted drugs

In the last decade, tremendous advances have revolutionized the biological landscape of acute myeloid leukemia (AML). The application of sophisticated molecular biology tools has unveiled a plethora of (epi)genomic and transcriptomic alterations, some of which have been recently recognized in pre-leukemic conditions (e.g. clonal hematopoiesis of indeterminate potential - CHIP (1)) with implications on AML ontogenesis and prognosis, paving the way for targeted approaches. The most recent diagnostic and prognostic schemes have included these latest advances (2), further improving the clinical management of AML patients.

The present Research Topic has provided a glimpse on some of these aspects, including: 1) new insights on AML biology, with the identification of novel transcriptomic signatures affecting prognosis, mechanistic description of pathogenic mutations (i.e. RAS) and oncogenic fusions (SET-CAN/NUP214) and the recent discoveries on leukemia stem cells (LSCs) at single-cell resolution; 2) new therapeutic perspectives in either induction (lower-dose chemotherapy + venetoclax or alternative chemotherapies for mixed phenotype acute leukemia – MPAL) or relapsed/refractory (R/R) AML (venetoclax + hypomethylating agents – HMA, targeted agent Selinexor, CAR-T cells). As a result, a balanced selection of articles spanning from AML biology and novel prognostic indicators to innovative therapeutic strategies has been selected for the readers.

Regarding AML biology, Villar et al. analyzed the transcriptome of 224 AML patients > 65 years-old at diagnosis treated in the Spanish PETHEMA-FLUGAZA clinical trial in order to identify new prognostic biomarkers in this population. They identified a specific transcriptomic signature for high-risk patients, revealing that low expression of *B7H3* gene with high expression of *BANP* gene identifies a subset with a more favorable prognosis surviving more than 12 months. This result was further validated in the BEAT AML cohort.

Zhang et al. focused on autophagy, since beside being known as a natural cellular response to a wide spectrum of cellular processes, it is debated whether it might have a pathogenic role in leukemias and particularly in AML. The authors found >100 differentially expressed genes (DEGs) related to autophagy between AML and healthy controls. Next, they selected 12 of these genes and developed a prognostic model predictive for survival of AML patients in both TCGA data and independent AML cohorts from GEO databases.

Wan et al. in a first-time approach based on the differential expression of lysosome-related genes in AML identified two different subtypes: cluster1 showed longer overall survival (OS) and stronger immune infiltration compared to cluster2. The pivotal differential genes between the two clusters are *SYK*, whose pro-carcinogenic mechanism relies in the promotion of AML cell survival and drug resistance (3), and *TLR4* which is thought to modulate lysosomal function and is in turn degraded by lysosome themselves (4). Finally, a prognostic model consisting of six genes identified patients in a low-risk group who survived significantly longer than those in the high-risk group and had higher immune infiltration and stronger response to immunotherapy.

Further, Song et al. unravel the role of SET-CAN/NUP214 fusion in leukemia. This aberration mainly occurs in T-cell acute lymphoblastic leukemia (T-ALL) patients, but it has also been reported in other leukemias including AML, MPAL and B-ALL. Leukemias bearing this fusion often share common immunophenotypic markers such as: CD7, cCD3, CD34, CD33 and CD13. This supports a model where the transformation of SET-CAN/NUP214+ leukemia may occur in the early stage of myeloid or lymphocyte differentiation, and it may be related to the inhibition of differentiation of primitive progenitor cells by the fusion gene. Patients with SET-CAN/NUP214 fusion usually exhibit resistance to chemotherapy, including glucocorticoids in the early stages of induction therapy, however the overall CR rate is not affected.

In another original paper, Liang et al. focus on the pathogenic role of RAS mutations in AML, and their impact on cell metabolism. In fact, RAS gene mutations are prevalent in AML, and the RAS signaling pathway is closely related to many metabolic pathways. By using a Ba/F3 cell line model transduced with NRAS^Q61K^ and KRAS^G12V^ mutations, the authors conducted a DEG analysis between mutant and wild-type cell lines. They found 1899 DEGs, of which 1089 were related to metabolic pathways, particularly the *DGKzeta* and *PLA2G4A* genes in the glycerophospholipid metabolism pathway were significantly upregulated. These findings may contribute to new precision therapy strategies and the development of new therapeutic drugs for AML.

In a comprehensive review, Zhou et al. provide a detailed summary of single-cell sequencing strategies in AML. These techniques have revolutionized our understanding of AML pathogenesis by enabling high-resolution interrogation of the cellular heterogeneity in the AML ecosystem. The authors focus on the identification of different leukemia stem cells (LSCs), T-cell subpopulations displaying exhausted phenotypes permissive towards AML, and pinpoint novel actionable liabilities. Such targets include: LGALS1 (promoter of resistance to therapy), CD52 and CD47 (expressed on the quiescent LSCs), CSF1R and CD86 (highly expressed on LSCs). These latter two have been proved to be effective CAR-T targets in preclinical evaluation (5).

On the clinical side, this Research Topic provides both Original and Review articles highlighting different therapeutic strategies in AML. Several authors introduce novel combinations on real-life patients cohorts. Zhang et al. present a different induction strategy involving 3 days of cyclophosphamide and cytarabine plus low dose venetoclax in 25 newly diagnosed AML patients, achieving a 92% CR/Cri rate (all MRD-) and 79% overall survival at 12 months.

The Polish group of Karasek et al. elaborate on a first-line induction strategy using CLAG-M combination for mixed phenotype acute leukemia (MPAL) patients, an interesting approach for a disease still judged “orphan” due to its lineage ambiguity and elusive biology. The authors report an ORR of 73%, however responses need to be consolidated with allogeneic transplant to avoid relapses.

In the context of R/R AML, Chen et al. compared the outcome of AML patients relapsing after allogeneic transplant and treated with venetoclax + azacytidine (VEN+HMA) vs those undergoing intensive chemotherapy. ORR rates were not different between the two arms (60% vs 64%), leading to a median OS of 6.8 months for both arms. However, toxicity profile of VEN+HMA was more favorable, with fewer infections (17% vs 50%), thrombocytopenia (74% vs 95%) and acute graft-versus-host disease.

A Case Report by Sperotto et al. highlights the efficacy of CPX-351 (liposomal cytarabine and daunorubicin) as a salvage chemotherapy in a patient who developed a secondary AML (t-AML) 15 years after treatment for acute promyelocytic leukemia (all-trans-retinoic acid and chemotherapy). The patient achieved a complete remission, underwent an allogeneic transplant and was alive after 2 years of follow-up.

Despite these and other therapeutic interventions, several AML patients still fail and/or relapse after first-line chemotherapy; these are the ones experiencing the worst outcome. Identifying novel treatment options for this subgroup of patients is an unmet clinical need. Exportin-1 (XPO-1) is usually overexpressed in various tumors, including relapsed AML. Selinexor, an inhibitor of XPO1, effectively promotes nuclear retention and functional activation of tumor suppressor proteins, thereby inducing apoptosis in cancer cells. In their original Report, Zhang et al. describe a novel combination of Selinexor plus decitabine and half-dose CAG chemotherapy in an elderly patient with R/R AML, leading to complete remission and good tolerance.

Lastly, among the novel promising treatments, CAR-T cells are still under evaluation in AML but they might eventually take their primal role as in other onco-hematological diseases. A Review on the role of CAR-T cells by Wei et al. in AML and T-ALL provides a great summary of the therapeutic efficacy of adoptive cell strategies in these two entities. CAR-T cells targeting CD5 and CD7 for T-ALL and CD123, CD33, and CLL1 for AML show promising efficacy and safety profiles in clinical trials.
